# The Bookworld of Medicine and Science

**Published:** 1901-03-23

**Authors:** 


### THE BOOKWORLD OF MEDICINE AND SCIENCE.

MOSQUITOS AND MALARIA: A SUMMARY OF KNOWLEDGE ON THE SUBJECT UP TO DATE, WITH AN ACCOUNT OF THE NATURAL HISTORY OF SOME MOSQUITOS. By CUTHBERT CHRISTY, M.B. and C.M. (Edin.). With six full-page plates. (London: Sampson Low, Marston, and Co. 1900. Price 6s. net.)

THIS is a very clearly written account of the now well-known mosquito theory of malaria. It is written in an easy and somewhat popular style, and the story of this great discovery is very well set out. To medical men perhaps the chief utility of the book will lie in the fact that it gives the dates and shows the sequence of the various investigations which led up to our recent knowledge on the subject, for events have followed each other so rapidly that one is all too apt to forget the intermediate steps. The most important and probably the most useful portion of the book is that which deals with the natural history of some of the many forms of mosquitos. This part of the book is well illustrated with some very clearly-drawn plates, showing the various stages in the life history of these creatures; and seeing how very largely we must depend upon an extended knowledge of the habits and modes of life of the mosquito tribe in any efforts we may make to control a disease in the propagation of which they take so large a part as they do in the spread of malaria, this part of the work will be of considerable value. The book is a very complete summary of our knowledge—up to a certain point—of a very rapidly advancing department of knowledge, with which, however, it must be confessed, it is not easy for book-makers to keep pace.

PHOTOGRAPHIC ATLAS OF THE DISEASES OF THE SKIN. By GEORGE HENRY FOX, A.M., M.D., Clinical Professor of Diseases of the Skin, College of Physicians and Surgeons, New York; Consulting Dermatologist to the Board of Health, New York City. Parts I., II., and III. To be completed in sixteen monthly parts. (Philadelphia and London: J. B. Lippincott Co. 1900. Price 6s. each part, net.)

THIS is not a mere atlas, for it contains also in each part a few pages of letterpress descriptive of the diseases depicted in the plates, and giving many useful indications in regard to their treatment. This "literary" portion of the work is really very interesting, and in many ways instructive. It is far from being a mere comment on the plates, teeming as it does with therapeutic suggestions, many of which are of considerable value, and it promises as the work proceeds to become a fairly complete treatise on the management of diseases of the skin. This real centre and object of the atlas is, however, to present an accurate pictorial demonstration of the principal diseases of the skin, and no one who examines the beautiful pictures it contains can doubt that this side of the undertaking has been accomplished with great success. The book is called a "Photographic Atlas," and the plates (of which there are five in each part, some containing more than one figure) are apparently reproductions of photographs taken direct from the cases depicted. They are, however, coloured, or rather tinted, so that they give a very good idea of the conditions represented, although it must be confessed that, as with all coloured photographs, the tendency to blackness in the shadows detracts somewhat from the vraisemblance. Still, no one can mistake what is meant, even in regard to the finer details. Take it altogether, we have nothing but praise for this atlas, so far as it has gone; and we have no doubt it will be found a most useful addition to a practitioner's library.

